# Supplementary material for: Commissureless acts as a substrate adapter in a conserved Nedd4 E3 ubiquitin ligase pathway to promote axon growth across the midline
Source: bioRxiv. 2024 Oct 31:2023.10.13.562283. Originally published 2023 Oct 17. Preprint. [Version 2] doi: 10.1101/2023.10.13.562283 (PMC10614773; doi:10.1101/2023.10.13.562283)
Supplement: Supplement 2 [file NIHPP2023.10.13.562283v2-supplement-2.pdf]

bioRxiv preprint doi: https://doi.org/10.1101/2023.10.13.562283; this version posted October 31, 2024. The copyright holder for this preprint (which was not certified by peer review) is the author/funder, who has granted bioRxiv a license to display the preprint in perpetuity. It is made available under aCC-BY 4.0 International license.

Supplementary figures and legends

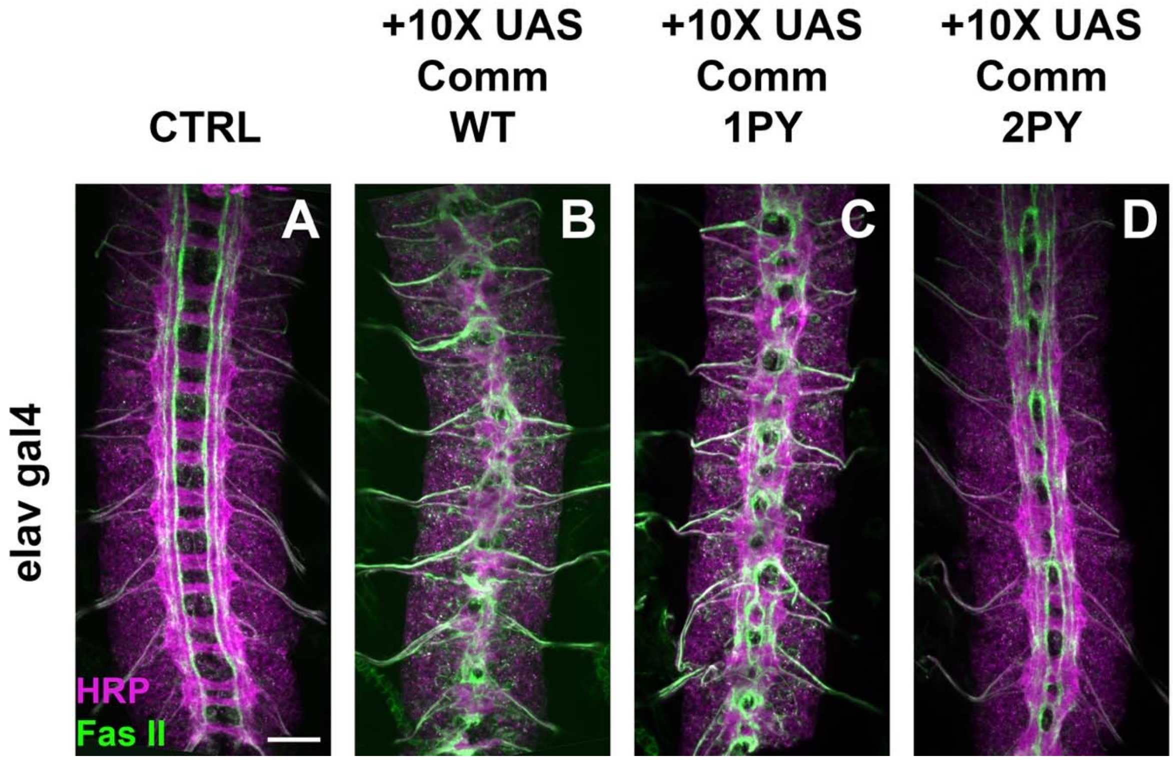

Figure S1: Driving high levels of Comm, including those lacking functional PY motifs, induces ectopic midline crossing in ipsilateral neurons. A-D) Nerve cords of embryos expressing no Comm (A), or 10X UAS transgenes for Comm WT(B) Comm 1PY (C) and Comm 2PY (D). Embryos are stained with the pan neuronal marker HRP (green) and an antibody against FasII (magenta), which labels a population of ipsilateral neurons. Scale bar represents 20μM.

bioRxiv preprint doi: https://doi.org/10.1101/2023.10.13.562283; this version posted October 31, 2024. The copyright holder for this preprint (which was not certified by peer review) is the author/funder, who has granted bioRxiv a license to display the preprint in perpetuity. It is made available under aCC-BY 4.0 International license.

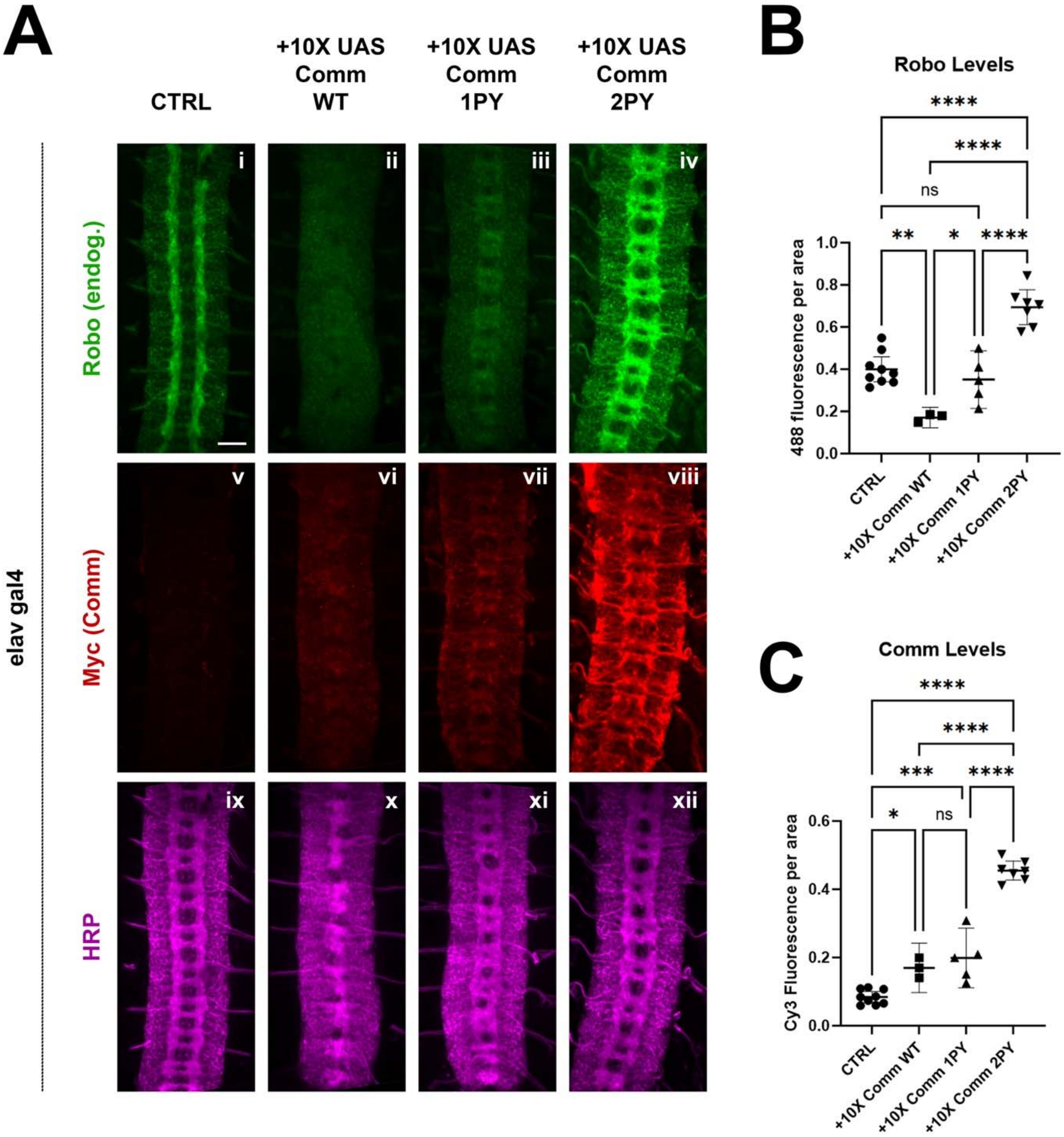

Figure S2: Robo expression in 10XUAS Comm transgenic flies

Stage 15-16 embryos expressing no Comm or 10X UAS Comm-myc variants under the pan neuronal elav gal4 driver. Embryos are stained for endogenous Robo1 (i-iv), Myc (v-viii), and pan-neuronal marker HRP (ix-xii). Scale bar represents 20μM. B) Quantification of endogenous Robo1 levels in stage 15-16 embryos expressing Comm variants under the elav gal4 driver. Robo1 levels were measured by creating a mask of the axonal scaffold, measuring 488

bioRxiv preprint doi: https://doi.org/10.1101/2023.10.13.562283; this version posted October 31, 2024. The copyright holder for this preprint (which was not certified by peer review) is the author/funder, who has granted bioRxiv a license to display the preprint in perpetuity. It is made available under aCC-BY 4.0 International license.

fluorescence within the scaffold, and dividing by scaffold area. C) Quantification of Comm levels in stage 15-16 embryos expressing Comm variants under the *elavGal4* driver. Comm levels were measured by creating a mask of the whole nerve cord, measuring Cy3 fluorescence within the nerve cord, and dividing by nerve cord area. For B and C, groups were compared using ANOVA with Tukey's post-hoc test. ** $p<0.01$ **** $p<0.0001$. Error bars represent 95% confidence intervals around the mean. Each data point represents one embryo.

bioRxiv preprint doi: https://doi.org/10.1101/2023.10.13.562283; this version posted October 31, 2024. The copyright holder for this preprint (which was not certified by peer review) is the author/funder, who has granted bioRxiv a license to display the preprint in perpetuity. It is made available under aCC-BY 4.0 International license.

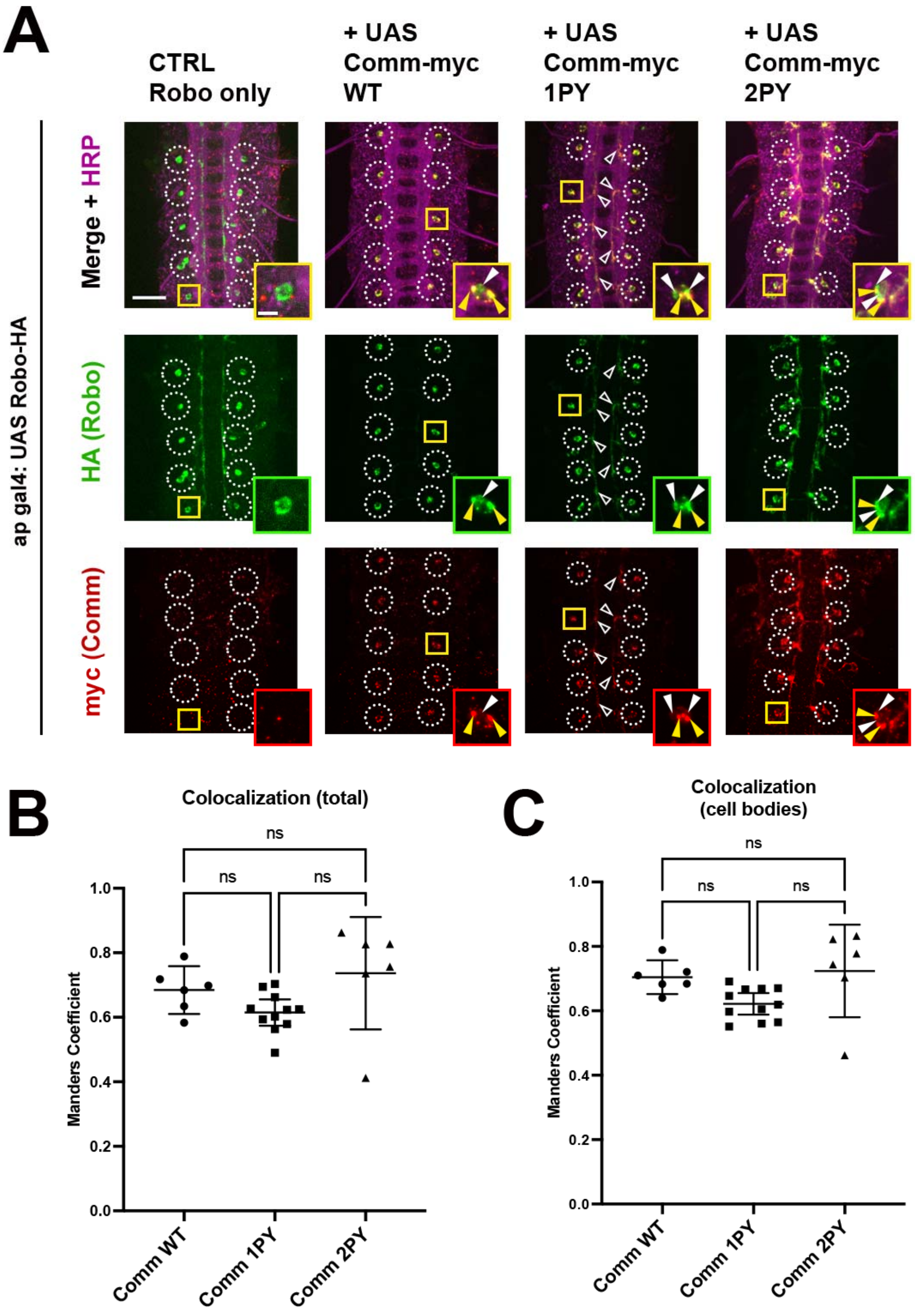

bioRxiv preprint doi: https://doi.org/10.1101/2023.10.13.562283; this version posted October 31, 2024. The copyright holder for this preprint (which was not certified by peer review) is the author/funder, who has granted bioRxiv a license to display the preprint in perpetuity. It is made available under aCC-BY 4.0 International license.

Figure S3: Robo colocalization with Comm is unaffected by mutations in Comm PY motifs

A) Stage 16-17 embryos expressing HA-Robo and WT or PY-mutant Comm myc variants under the *apGal4* driver. Embryos are stained with HA (Robo), Myc (Comm), and HRP (pan neuronal marker). Cell bodies are outlined with white circles except those that are enlarged in inset images, which are outlined with yellow squares. Within inset images, Robo/Comm co-positive puncta are indicated with yellow arrowheads and puncta containing Robo alone are indicated with white arrowheads. Axonal expression of Robo and Comm is indicated with hollow arrowheads. Scale bar represents 20µM in the large image and 5µM in the inset image. B) Total colocalization of Robo and Comm. The proportion of areas of Robo expression that were also positive for Comm was calculated via Manders coefficient within a mask created of the area of Robo expression using smoothened images from the Robo channel, which contain both cell bodies and axons. C) Colocalization of Comm and Lamp1 within cell bodies. The proportion of areas of Robo expression that were also positive for Comm was calculated via Manders coefficient within a mask created of cell bodies using smoothened images from the Robo channel, manually edited to remove axons. For B and C, colocalization across conditions was compared via ANOVA with Tukey’s post hoc test (* $p<0.05$, ** $p<0.01$). Error bars represent 95% confidence intervals around the mean. Each data point represents one embryo.

bioRxiv preprint doi: https://doi.org/10.1101/2023.10.13.562283; this version posted October 31, 2024. The copyright holder for this preprint (which was not certified by peer review) is the author/funder, who has granted bioRxiv a license to display the preprint in perpetuity. It is made available under aCC-BY 4.0 International license.

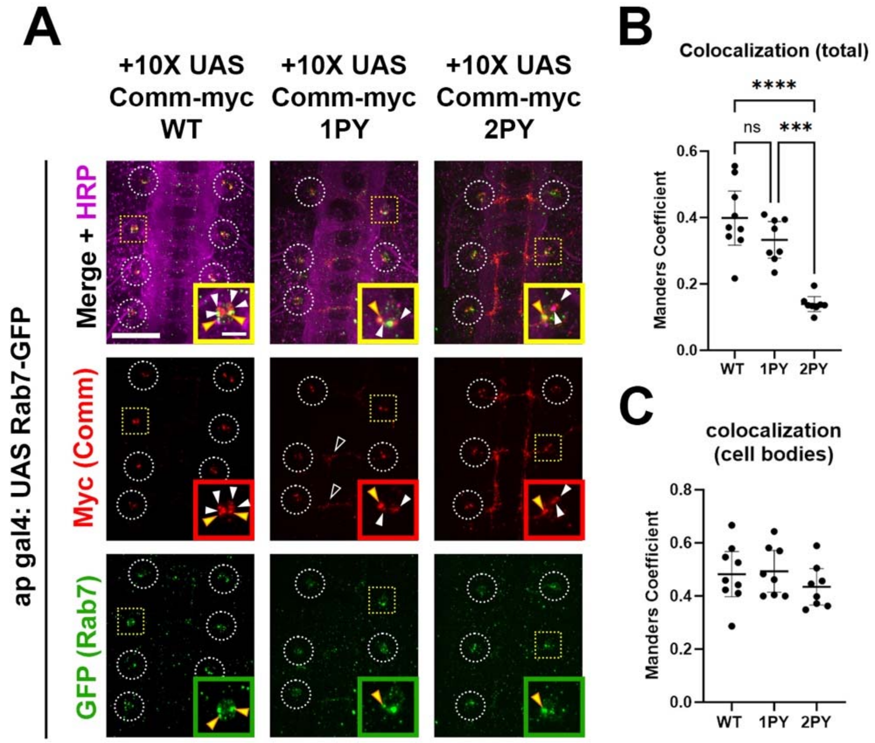

Figure S4: Colocalization of Comm with the late endosomal marker Rab 7

A) Stage 17 embryos expressing Rab7-GFP and WT or PY-mutant Comm myc variants under the *apGal4* driver. Embryos are stained with GFP (Rab7), Myc (Comm), and HRP (pan neuronal marker). Cell bodies are outlined with white circles except those that are enlarged in inset images, which are outlined with yellow squares. Within inset images, Comm/Rab7 co-positive puncta are indicated with yellow arrowheads and puncta containing Comm alone are indicated with white arrowheads. Scale bar represents 20μM. B) Total colocalization of Comm and Rab7. Proportion of areas of Comm expression that were also positive for Rab7 was calculated via pearson's coefficient within a mask created of the area of Comm expression using smoothened images from the Comm channel. C Colocalization of Comm and Rab7 within cell bodies. Proportion of areas of Comm expression that were also positive for Rab7 was calculated via pearson's coefficient within a mask created of cell bodies using smoothened images from the Lamp1 channel. For B and C, colocalization across conditions was compared via ANOVA (*

bioRxiv preprint doi: https://doi.org/10.1101/2023.10.13.562283; this version posted October 31, 2024. The copyright holder for this preprint (which was not certified by peer review) is the author/funder, who has granted bioRxiv a license to display the preprint in perpetuity. It is made available under aCC-BY 4.0 International license.

p<0.05, ** p<0.01). Error bars represent 95% confidence intervals around the mean. Each data point represents one embryo.

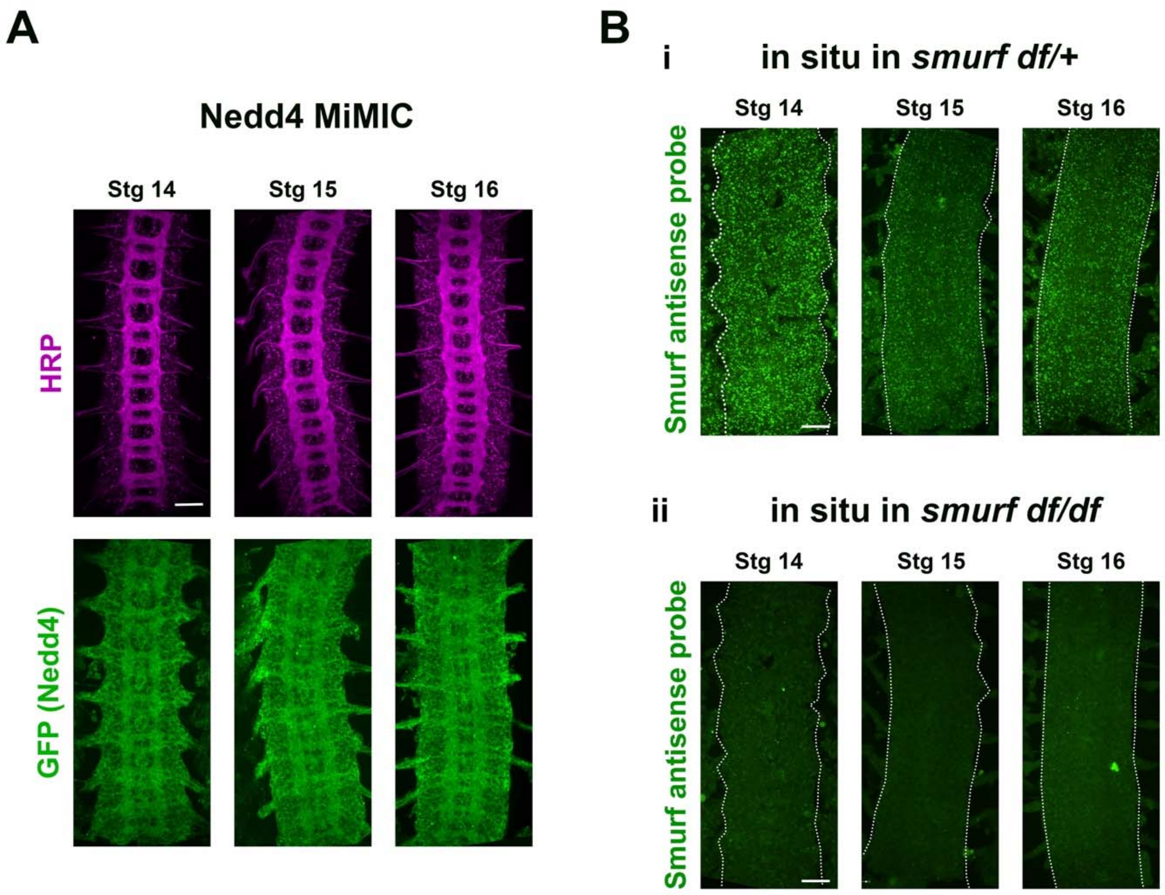

Figure S5: Nedd4 and Smurf are expressed in the nerve cord during midline crossing

A) Nerve cords of Stage 14-16 Nedd4 MiMIC embryos, in which endogenous Nedd4 protein is tagged with GFP. Nedd4 expression was visualized using an antibody against GFP and the axon scaffold was visualized with HRP. B) In situ using an antisense probe against Smurf in Stage 14-16 embryos heterozygous (i) or homozygous (ii) for a chromosomal deletion covering the Smurf locus. Nerve cords are outlined with dotted lines. All scale bars represent 20μM.

bioRxiv preprint doi: https://doi.org/10.1101/2023.10.13.562283; this version posted October 31, 2024. The copyright holder for this preprint (which was not certified by peer review) is the author/funder, who has granted bioRxiv a license to display the preprint in perpetuity. It is made available under aCC-BY 4.0 International license.

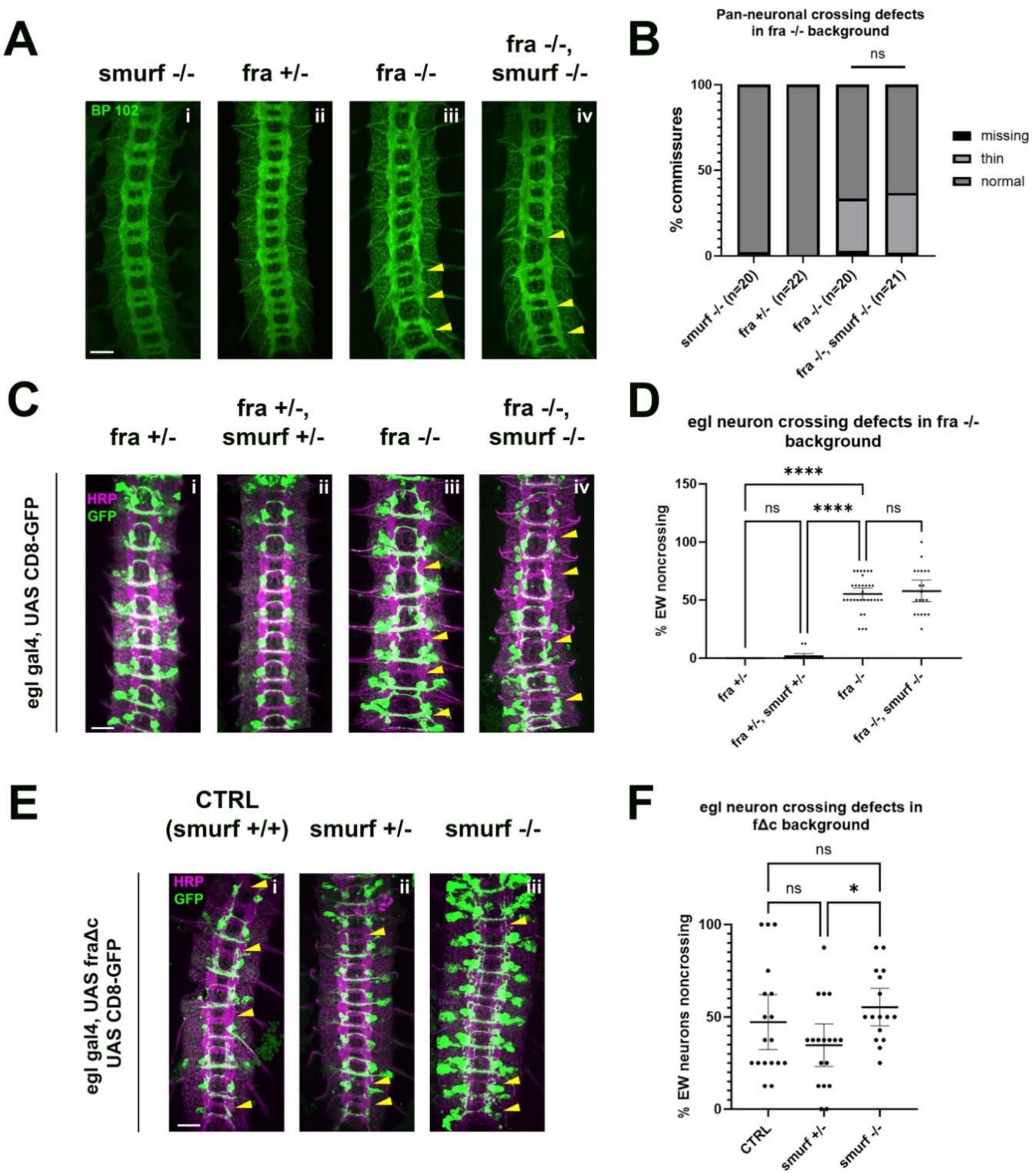

Figure S6: Smurf does not appear to be required for midline crossing *in vivo*

A-B) Loss of *smurf* does not enhance pan neuronal crossing defects in a *fra-/-* background. A) Stage 15-16 embryos with various combinations of *wild-type* and null *fra* and *smurf* alleles, stained with pan-neuronal marker BP102 (green). Commissures with crossing defects (either thin or completely missing) are indicated with yellow arrowheads. B) Quantification of crossing

bioRxiv preprint doi: https://doi.org/10.1101/2023.10.13.562283; this version posted October 31, 2024. The copyright holder for this preprint (which was not certified by peer review) is the author/funder, who has granted bioRxiv a license to display the preprint in perpetuity. It is made available under aCC-BY 4.0 International license.

defects in embryos within the genotypes indicated in (A). Percentage of total crossing defects was compared across groups using ANOVA (** $p<0.01$, **** $p<0.0001$). N represents number of individual embryos. C-D) Loss of *smurf* fails to enhance EW crossing defects in a fra -/- background. C Stage 15/16 embryos with various combinations of wt and null fra and nedd4 alleles, expressing GFP under the eg gal4 driver. Embryos are stained with pan-neuronal marker HRP, and GFP to visualize eg neurons. Commissures with EW crossing defects are indicated with arrowheads. D) Quantification of crossing defects in embryos within the genotypes indicated in C. Percentage of crossing defects was compared across groups using ANOVA (** $p<0.01$, **** $p<0.0001$). Each data point represents an individual embryo and error bars represent 95% confidence intervals around the mean. E-F) Loss of *smurf* fails to enhance EW crossing defects in the FraΔc background. E) Embryos with *smurf* mutations in FraΔc background. F) Quantification of EW crossing defects for the genotypes shown in E. Percentage of crossing defects was compared across groups using ANOVA (** $p<0.01$, **** $p<0.0001$). Each data point represents an individual embryo and error bars represent 95% confidence intervals around the mean. All scale bars represent 20μM.

bioRxiv preprint doi: https://doi.org/10.1101/2023.10.13.562283; this version posted October 31, 2024. The copyright holder for this preprint (which was not certified by peer review) is the author/funder, who has granted bioRxiv a license to display the preprint in perpetuity. It is made available under aCC-BY 4.0 International license.

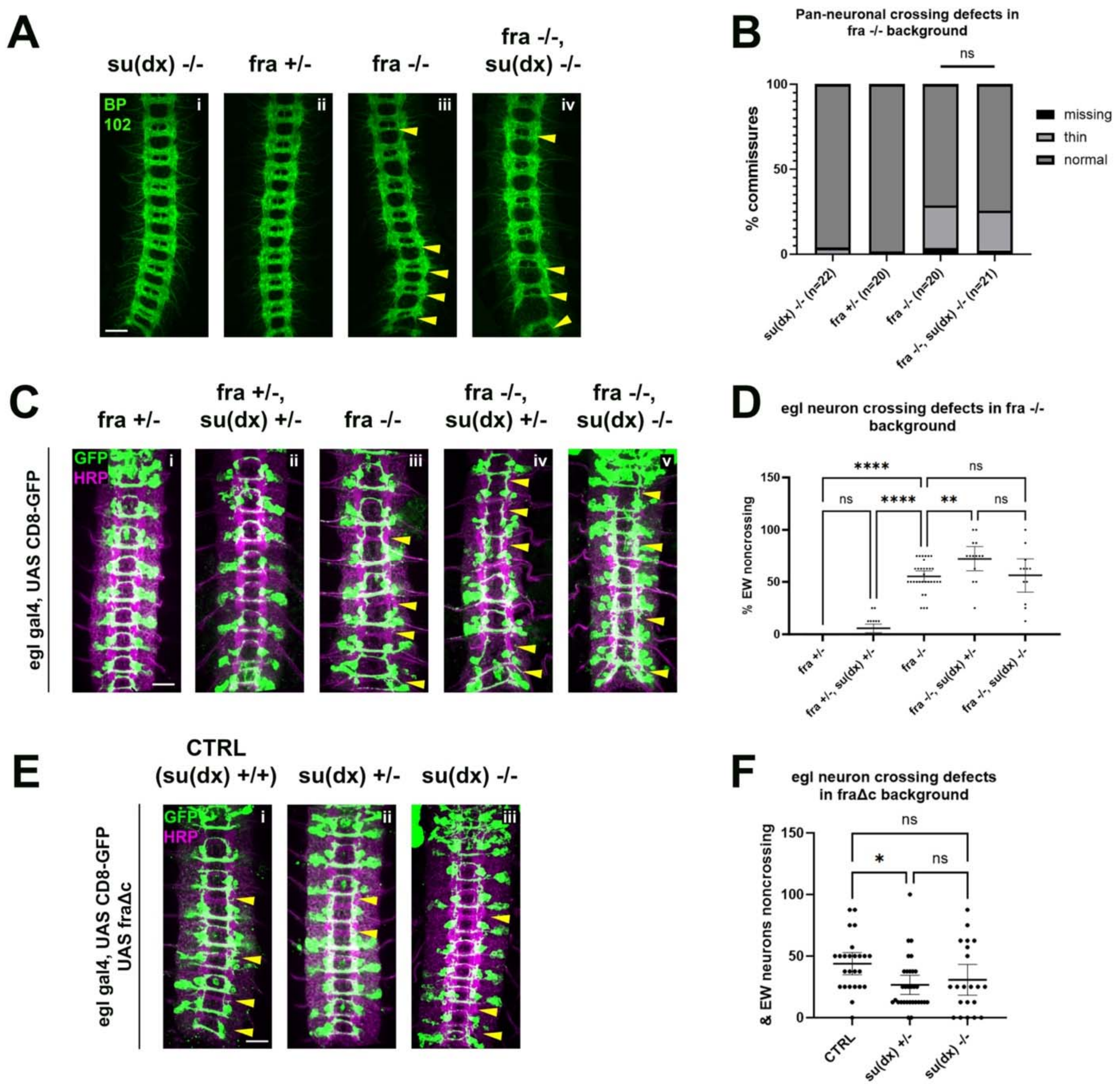

Figure S7: Su(dx) does not appear to be required for midline crossing *in vivo*

A-B) Loss of *Su(dx)* fails to enhance pan neuronal crossing defects in a fra-/- background. A) Stage 15/16 embryos with various combinations of *wild-type* and null *fra* and *Su(dx)* alleles, stained with pan-neuronal marker BP102 (green). Commissures with crossing defects (either thin or completely missing) are indicated with yellow arrowheads. B) Quantification of crossing defects in embryos within the genotypes indicated in A. Percentage of total crossing defects was compared across groups using ANOVA (** p<0.01, **** p<0.0001). N represents number of

bioRxiv preprint doi: https://doi.org/10.1101/2023.10.13.562283; this version posted October 31, 2024. The copyright holder for this preprint (which was not certified by peer review) is the author/funder, who has granted bioRxiv a license to display the preprint in perpetuity. It is made available under aCC-BY 4.0 International license.

individual embryos. C-D) Loss of *Su(dx)* fails to enhance EW crossing defects in a *fra -/-* background. C Stage 15/16 embryos with various combinations of *wild-type* and null *fra* and *nedd4* alleles, expressing GFP under the eg gal4 driver. Embryos are stained with pan-neuronal marker HRP, and GFP to visualize eg neurons. Commissures with EW crossing defects are indicated with arrowheads. D) Quantification of crossing defects in embryos within the genotypes indicated in C. Percentage of crossing defects was compared across groups using ANOVA (** $p<0.01$, **** $p<0.0001$). Each data point represents an individual embryo and error bars represent95% confidence intervals around the mean. E-F) Loss of *Su(dx)* fails to enhance EW crossing defects in the FraΔc background. E) Embryos with *Su(dx)* mutations in FraΔc background. F) Quantification of EW crossing defects for the genotypes shown in E. Percentage of crossing defects was compared across groups using ANOVA (** $p<0.01$, **** $p<0.0001$). Each data point represents an individual embryo and error bars represent95% confidence intervals around the mean. All scale bars represent 20μM**.**

bioRxiv preprint doi: https://doi.org/10.1101/2023.10.13.562283; this version posted October 31, 2024. The copyright holder for this preprint (which was not certified by peer review) is the author/funder, who has granted bioRxiv a license to display the preprint in perpetuity. It is made available under aCC-BY 4.0 International license.

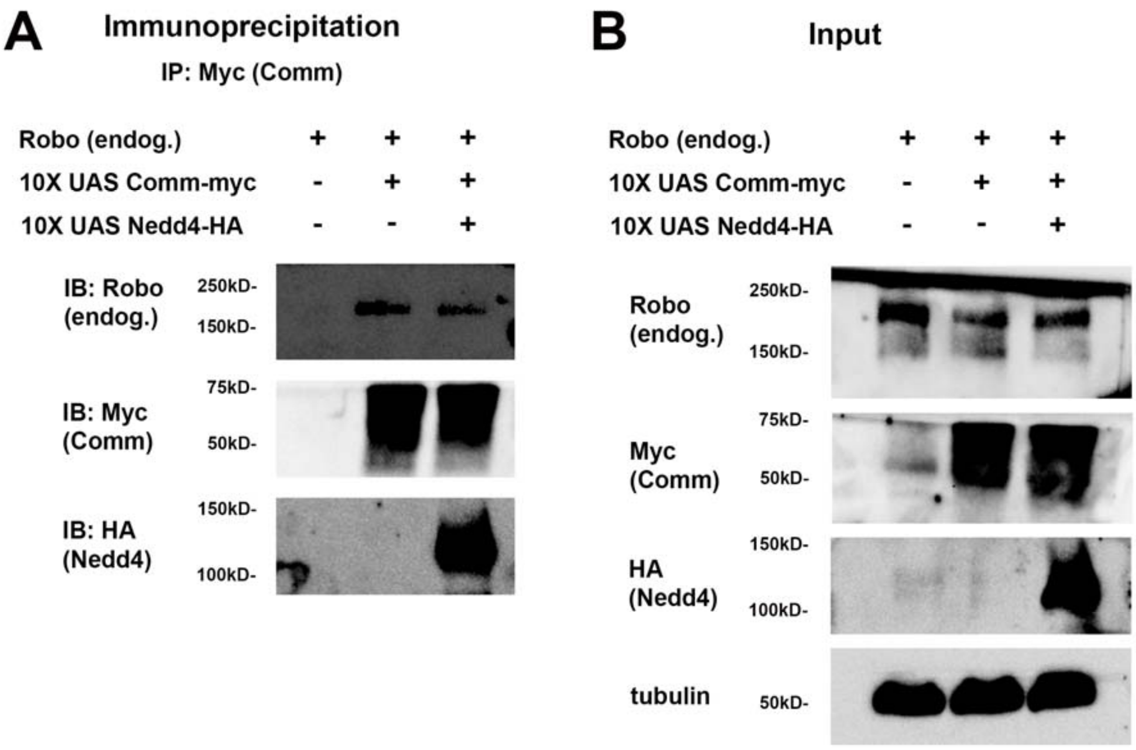

Figure S8: Robo, Comm, and Nedd4 form a three-member complex in fly embryonic lysate

Immunoprecipitation (A) and input (B) of lysates of 24hpf embryos expressing WT Comm-myc, with or without Nedd4 HA, under the pan-neural *elav gal4* driver.

bioRxiv preprint doi: https://doi.org/10.1101/2023.10.13.562283; this version posted October 31, 2024. The copyright holder for this preprint (which was not certified by peer review) is the author/funder, who has granted bioRxiv a license to display the preprint in perpetuity. It is made available under aCC-BY 4.0 International license.

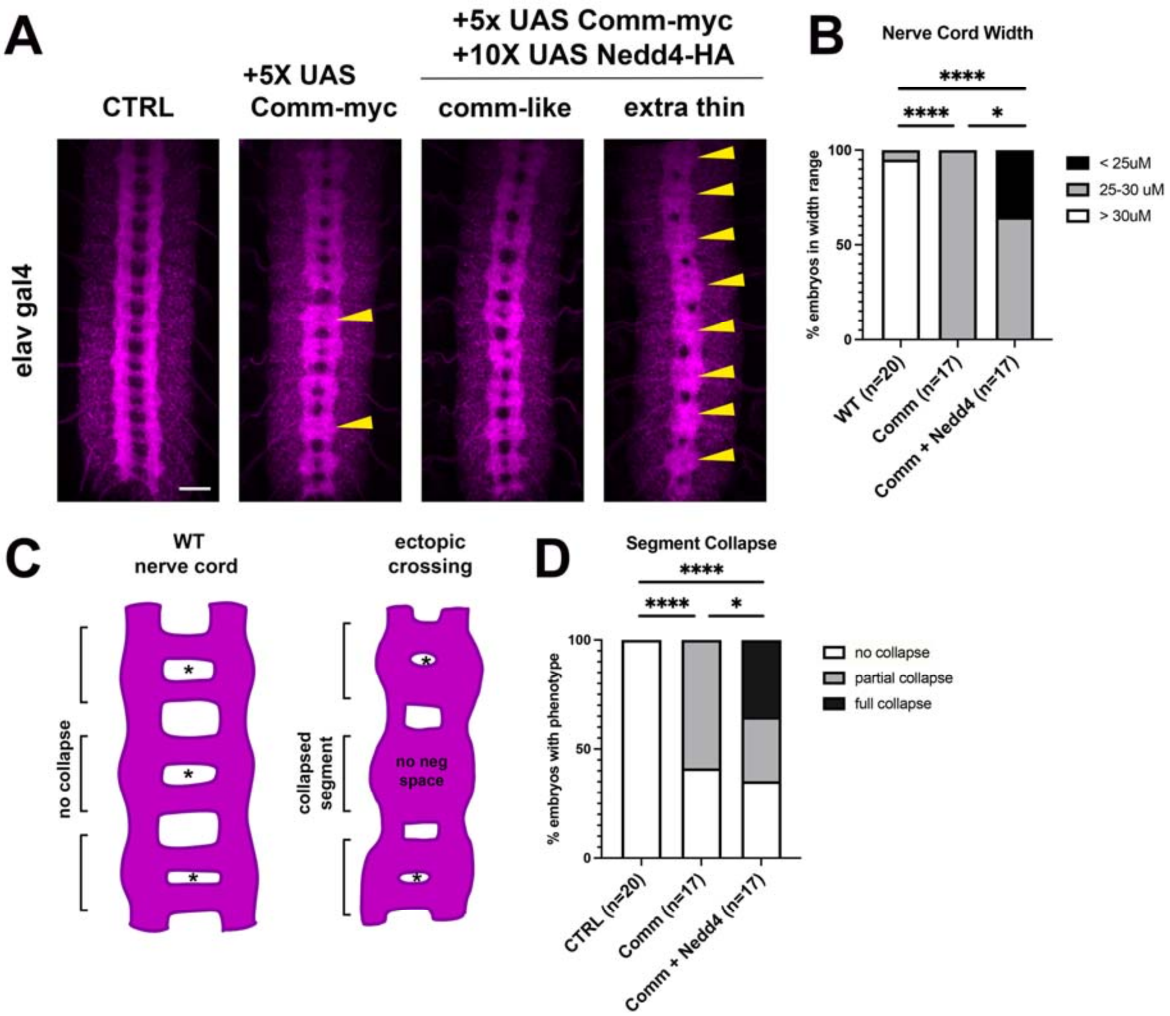

Figure S9: Nedd4 enhances ectopic crossing phenotype induced by Comm overexpression

A) Nerve cords of stage 16-17 embryos expressing WT Comm-myc with or without Nedd4-HA, under the pan neuronal *elav gal4* driver. Embryos are stained with the pan neuronal marker HRP. The width of a single body segment is marked with a white line. Scale bar represents 20uM. Collapsed segments, described further in C, are indicated with yellow arrowheads. B) Nerve cord width of stage 16-17 embryos expressing WT Comm-myc with or without Nedd4-HA, under the pan neuronal *elav gal4* driver. Nerve cord with is calculated by taking the average of the widths of the posterior eight body segments

bioRxiv preprint doi: https://doi.org/10.1101/2023.10.13.562283; this version posted October 31, 2024. The copyright holder for this preprint (which was not certified by peer review) is the author/funder, who has granted bioRxiv a license to display the preprint in perpetuity. It is made available under aCC-BY 4.0 International license.

at their widest points (the unit represented by a white line in A). Nerve cord widths were binned into three different phenotypic classes and distribution of phenotypes between groups was compared using Fisher's exact test with Freeman-Halton extension using raw counts per phenotypic class. C-D) Nedd4 enhances collapse of nerve cord segments induced by Comm overexpression. C) Diagram of *Drosophila* nerve cord axonal scaffolds from a WT embryo and one exhibiting some ectopic crossing. Nerve cord segments are indicated with brackets and negative space within the segments are indicated with asterisks. Nerve cord segments are considered collapsed when they have no negative space. D) Percentage of collapsed segments in stage 16-17 embryos expressing WT Comm-myc with or without Nedd4-HA, under the pan neuronal *elav gal4* driver. Percentage of collapsed holes is calculated using the following formula: (number of segments lacking negative space)/ (total segments) *100. The mask of the axonal scaffold used for this analysis was created by taking micrographs of HRP-stained nerve cords, applying a smoothing filter, and generating a thresholded image from this smoothened microscope photo. Embryos were binned into the following phenotypic categories: no collapse (0% collapse), partial collapse (0<x<100% collapse), and complete collapse (100% segments collapsed). Distribution of phenotypes between different genotypes was compared using Fisher's exact test with Freeman-Halton extension, using the raw count of embryos within each phenotypic class. Differences were considered significant if $p<0.05$. **** $p<0.0001$, * $p<0.05$.
