# Supplementary material for: Commissureless acts as a substrate adapter in a conserved Nedd4 E3 ubiquitin ligase pathway to promote axon growth across the midline
Source: bioRxiv. 2023 Oct 17:2023.10.13.562283. Preprint. [Version 1] doi: 10.1101/2023.10.13.562283 (PMC10614773; doi:10.1101/2023.10.13.562283)
Supplement: Supplementary file 1 [file NIHPP2023.10.13.562283v1-supplement-1.pdf]

bioRxiv preprint doi: https://doi.org/10.1101/2023.10.13.562283; this version posted October 17, 2023. The copyright holder for this preprint (which was not certified by peer review) is the author/funder, who has granted bioRxiv a license to display the preprint in perpetuity. It is made available under aCC-BY 4.0 International license.

Supplementary figures and legends

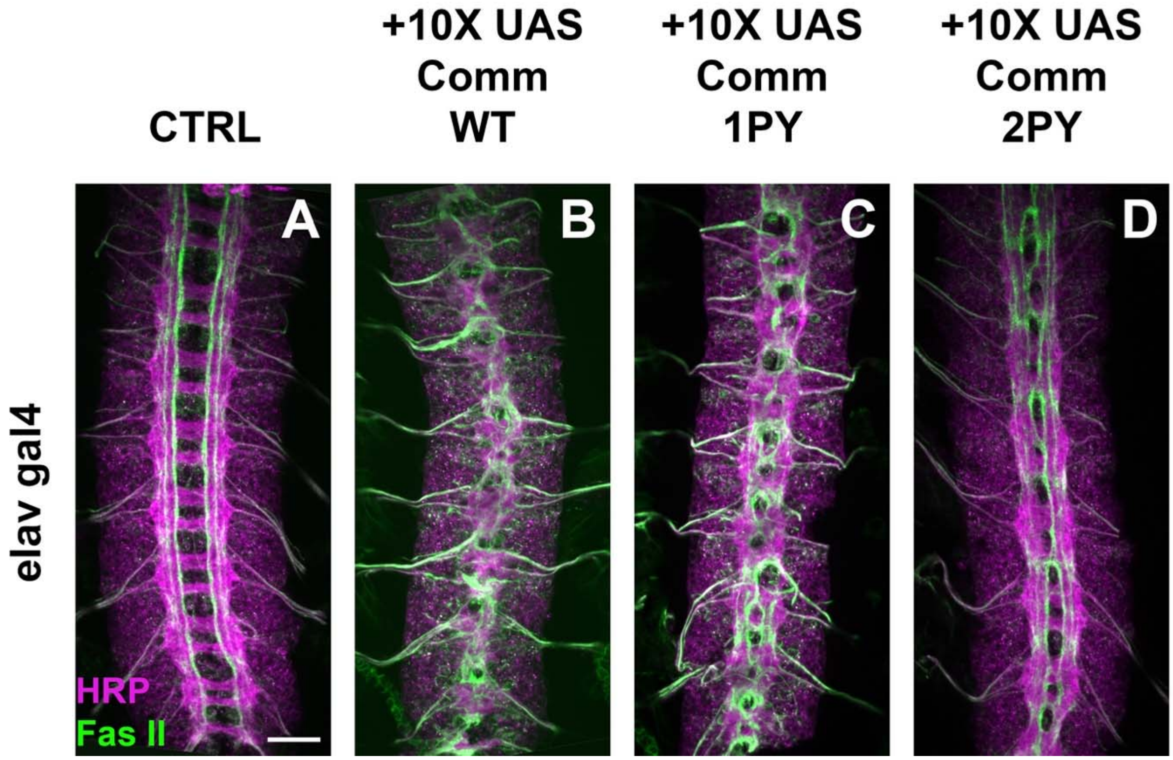

Figure S1: Driving high levels of Comm, including those lacking functional PY motifs, induces ectopic midline crossing in ipsilateral neurons. A-D) Nerve cords of embryos expressing no Comm (A), or 10X UAS transgenes for Comm WT(B) Comm 1PY (C) and Comm 2PY (D). Embryos are stained with the pan neuronal marker HRP (green) and an antibody against FasII (magenta), which labels a population of ipsilateral neurons. Scale bar represents 20μM.

bioRxiv preprint doi: https://doi.org/10.1101/2023.10.13.562283; this version posted October 17, 2023. The copyright holder for this preprint (which was not certified by peer review) is the author/funder, who has granted bioRxiv a license to display the preprint in perpetuity. It is made available under aCC-BY 4.0 International license.

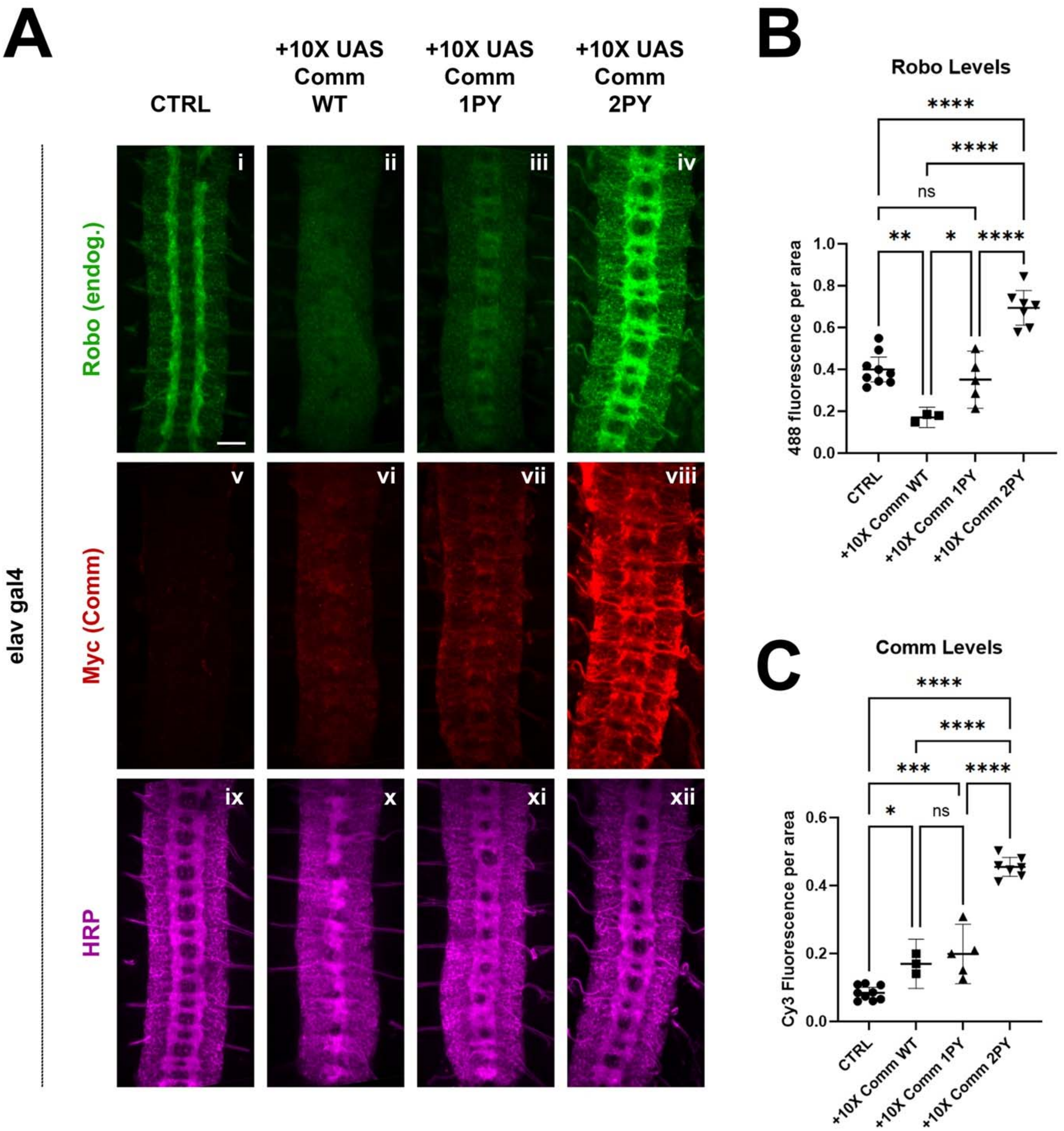

Figure S2: Robo expression in 10XUAS Comm transgenic flies. Stage 15-16 embryos expressing no Comm or 10X UAS Comm-myc variants under the pan neuronal elav gal4 driver. Embryos are stained for endogenous Robo1 (i-iv), Myc (v-viii), and pan-neuronal marker HRP (ix-xii). Scale bar represents 20μM. B) Quantification of endogenous Robo1 levels in stage 15-16 embryos expressing Comm variants under elav gal4 driver. Robo1 levels were measured by creating a mask of the axonal scaffold, measuring 488 fluorescence within the scaffold, and

bioRxiv preprint doi: https://doi.org/10.1101/2023.10.13.562283; this version posted October 17, 2023. The copyright holder for this preprint (which was not certified by peer review) is the author/funder, who has granted bioRxiv a license to display the preprint in perpetuity. It is made available under aCC-BY 4.0 International license.

dividing by scaffold area. C) Quantification of Comm levels in stage 15-16 embryos expressing Comm variants under the *elavGal4* driver. Comm levels were measured by creating a mask of the whole nerve cord, measuring Cy3 fluorescence within the nerve cord, and dividing by nerve cord area. For B and C, groups were compared using ANOVA with Tukey's post-hoc test. ** p<0.01 **** p<0.0001. Error bars represent 95% confidence intervals around the mean. Each data point represents one embryo.

bioRxiv preprint doi: https://doi.org/10.1101/2023.10.13.562283; this version posted October 17, 2023. The copyright holder for this preprint (which was not certified by peer review) is the author/funder, who has granted bioRxiv a license to display the preprint in perpetuity. It is made available under aCC-BY 4.0 International license.

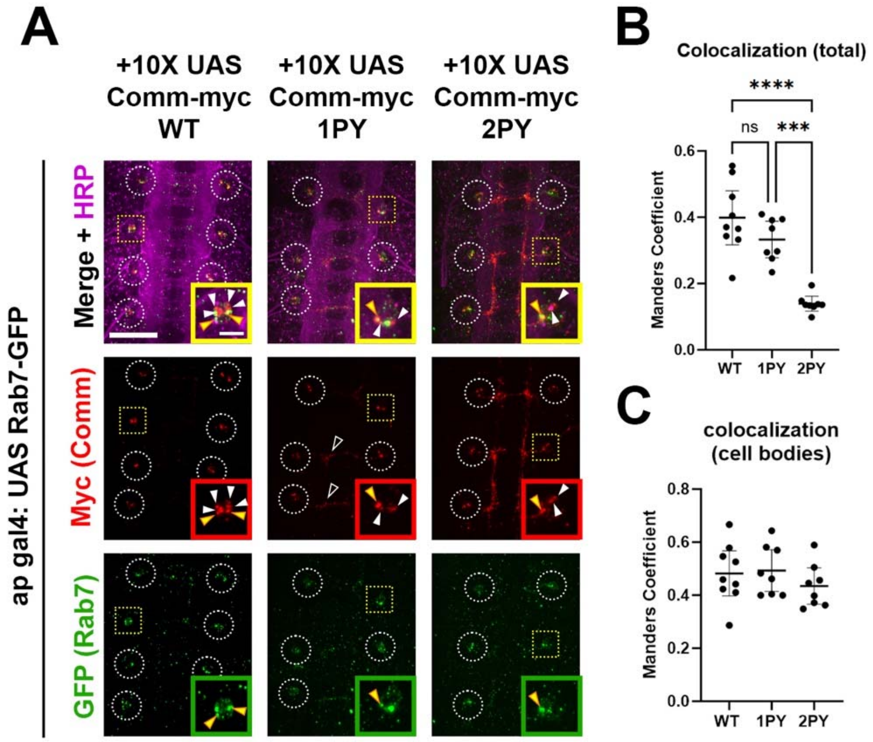

Figure S3: Colocalization of Comm with the late endosomal marker Rab 7. A) Stage 17 embryos expressing Rab7-GFP and WT or PY-mutant Comm myc variants under the *apGal4* driver. Embryos are stained with GFP (Rab7), Myc (Comm), and HRP (pan neuronal marker). Cell bodies are outlined with white circles except those that are enlarged in inset images, which are outlined with yellow squares. Within inset images, Comm/Rab7 co-positive puncta are indicated with yellow arrowheads and puncta containing Comm alone are indicated with white arrowheads. Scale bar represents 20μM. B) Total colocalization of Comm and Rab7. Proportion of areas of Comm expression that were also positive for Rab7 was calculated via pearson's coefficient within a mask created of the area of Comm expression using smoothened images from the Comm channel. C Colocalization of Comm and Rab7 within cell bodies. Proportion of areas of Comm expression that were also positive for Rab7 was calculated via pearson's coefficient within a mask created of cell bodies using smoothened images from the Lamp1 channel. For B and C, colocalization across conditions was compared via ANOVA (* $p<0.05$, **

bioRxiv preprint doi: https://doi.org/10.1101/2023.10.13.562283; this version posted October 17, 2023. The copyright holder for this preprint (which was not certified by peer review) is the author/funder, who has granted bioRxiv a license to display the preprint in perpetuity. It is made available under aCC-BY 4.0 International license.

p<0.01). Error bars represent 95% confidence intervals around the mean. Each data point represents one embryo.

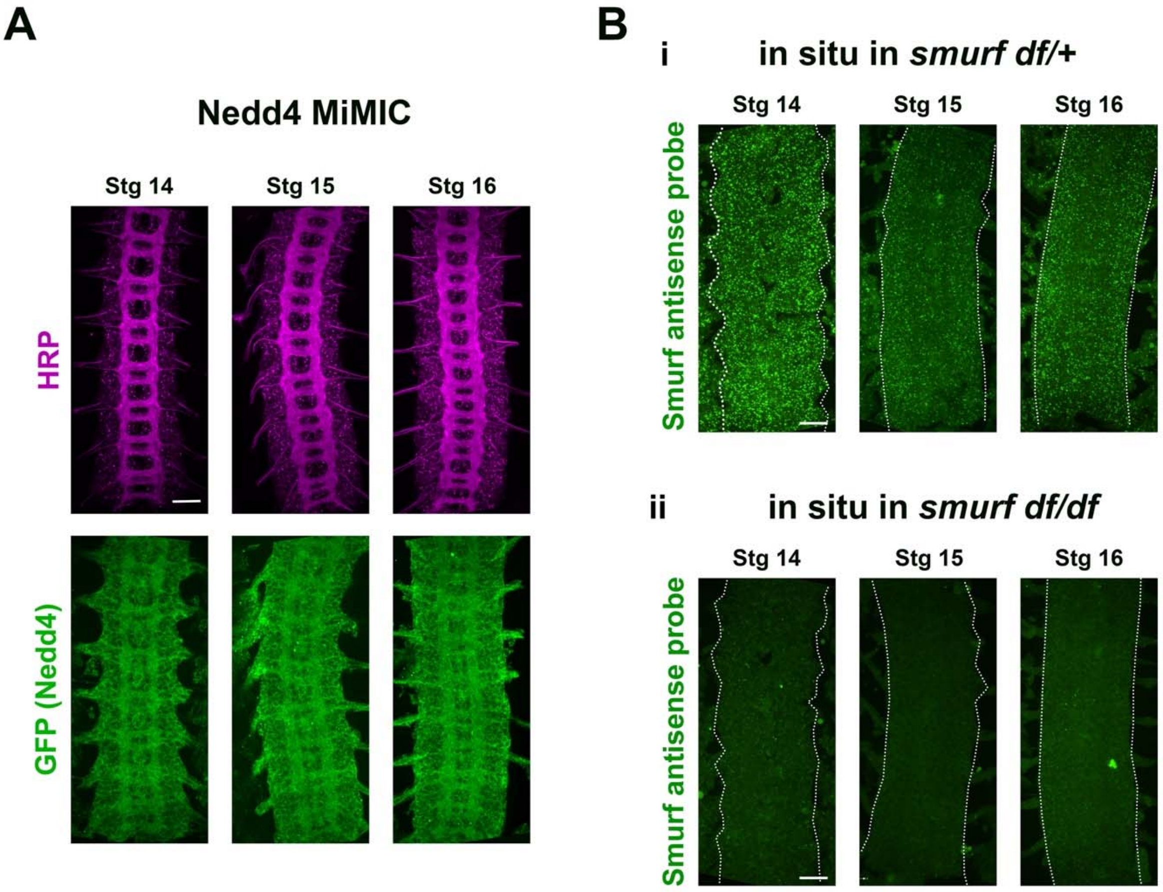

Figure S4: Nedd4 and Smurf are expressed in the nerve cord at developmental stages when midline crossing occurs. A. Nerve cords of Stage 14-16 Nedd4 MiMiC embryos, in which endogenous Nedd4 protein is tagged with GFP. Nedd4 expression was visualized using an antibody against GFP and the axon scaffold was visualized with HRP. B. In situ using an antisense probe against Smurf in Stage 14-16 embryos heterozygous (i) or homozygous (ii) for a chromosomal deletion covering the Smurf locus. Nerve cords are outlined with dotted lines. All scale bars represent 20μM.

bioRxiv preprint doi: https://doi.org/10.1101/2023.10.13.562283; this version posted October 17, 2023. The copyright holder for this preprint (which was not certified by peer review) is the author/funder, who has granted bioRxiv a license to display the preprint in perpetuity. It is made available under aCC-BY 4.0 International license.

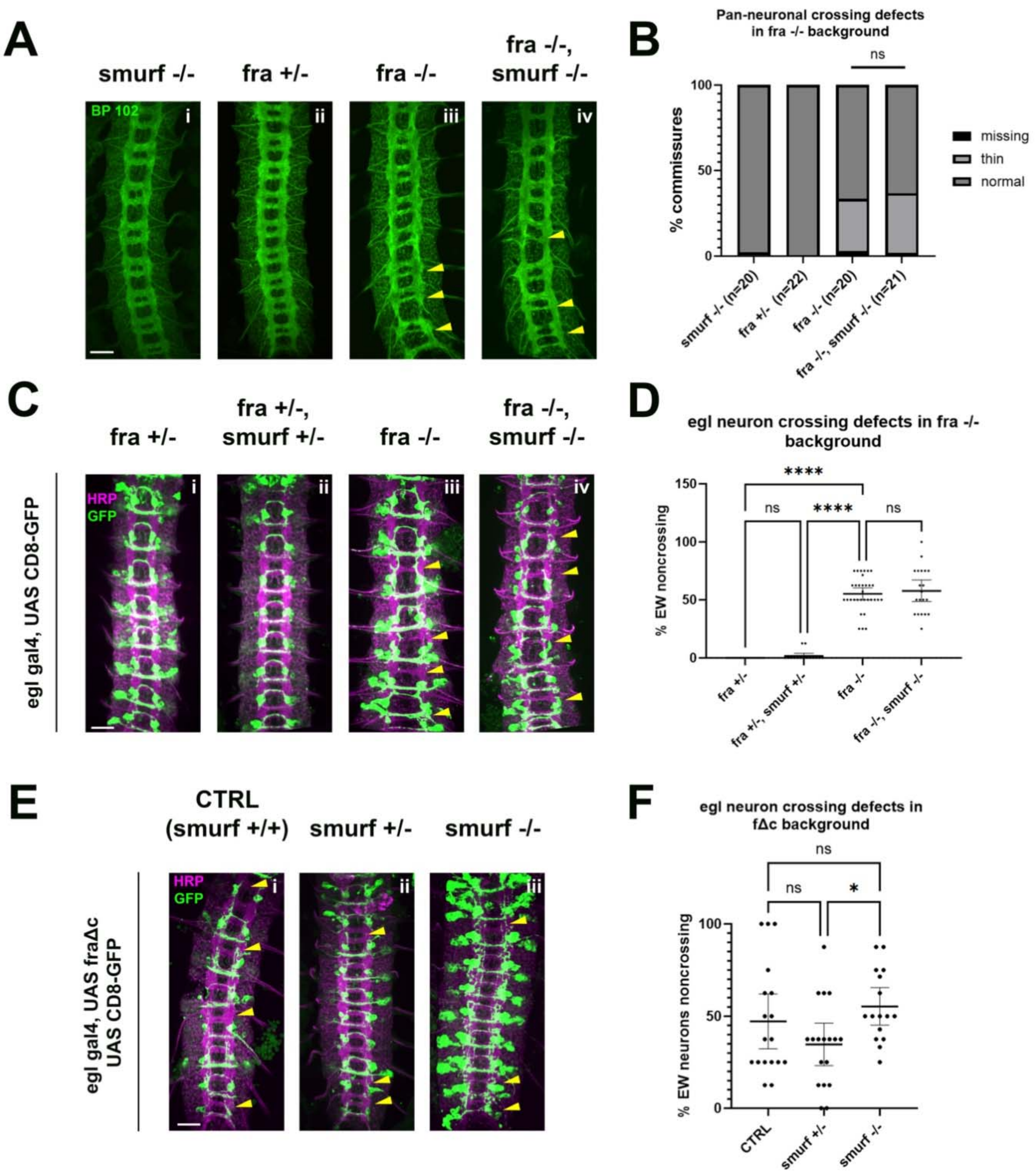

Figure S5: Smurf does not appear to be required for midline crossing *in vivo*. A-B) Loss of *smurf* does not enhance pan neuronal crossing defects in a *fra-/-* background. A) Stage 15-16 embryos with various combinations of *wild-type* and null *fra* and *smurf* alleles, stained with pan-neuronal marker BP102 (green). Commissures with crossing defects (either thin or completely missing) are indicated with yellow arrowheads. B) Quantification of crossing defects in embryos

bioRxiv preprint doi: https://doi.org/10.1101/2023.10.13.562283; this version posted October 17, 2023. The copyright holder for this preprint (which was not certified by peer review) is the author/funder, who has granted bioRxiv a license to display the preprint in perpetuity. It is made available under aCC-BY 4.0 International license.

within the genotypes indicated in (A). Percentage of total crossing defects was compared across groups using ANOVA (** $p<0.01$, **** $p<0.0001$). N represents number of individual embryos. C-D) Loss of *smurf* fails to enhance EW crossing defects in a fra -/- background. C Stage 15/16 embryos with various combinations of wt and null fra and nedd4 alleles, expressing GFP under the egl gal4 driver. Embryos are stained with pan-neuronal marker HRP, and GFP to visualize egl neurons. Commissures with EW crossing defects are indicated with arrowheads. D) Quantification of crossing defects in embryos within the genotypes indicated in C. Percentage of crossing defects was compared across groups using ANOVA (** $p<0.01$, **** $p<0.0001$). Each data point represents an individual embryo and error bars represent 95% confidence intervals around the mean. E-F) Loss of *smurf* fails to enhance EW crossing defects in the fraΔc background. E) Embryos with *smurf* mutations in fraΔc background. F) Quantification of EW crossing defects for the genotypes shown in E. Percentage of crossing defects was compared across groups using ANOVA (** $p<0.01$, **** $p<0.0001$). Each data point represents an individual embryo and error bars represent 95% confidence intervals around the mean. All scale bars represent 20μM.

bioRxiv preprint doi: https://doi.org/10.1101/2023.10.13.562283; this version posted October 17, 2023. The copyright holder for this preprint (which was not certified by peer review) is the author/funder, who has granted bioRxiv a license to display the preprint in perpetuity. It is made available under aCC-BY 4.0 International license.

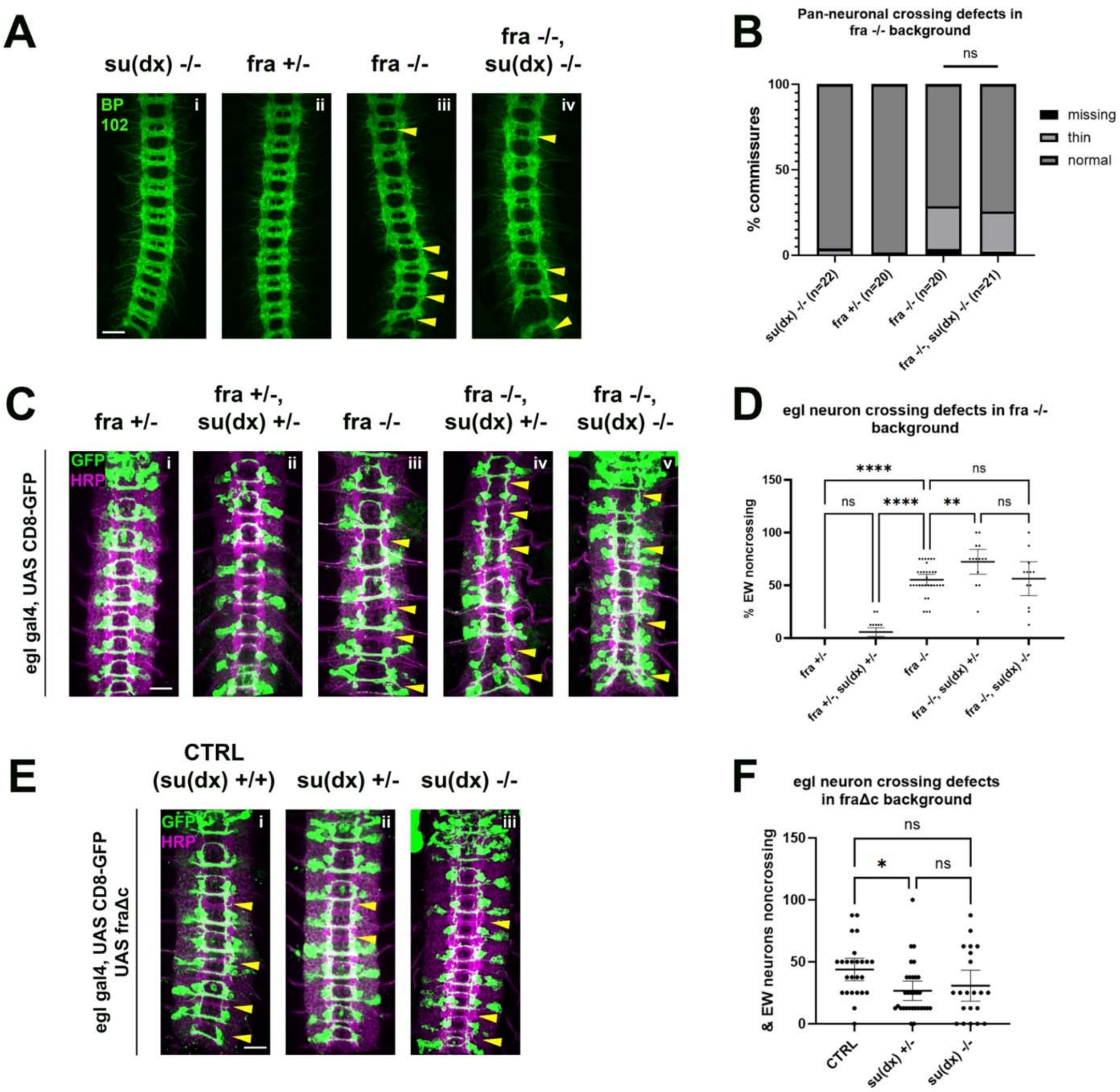

Figure S6: Su(dx) does not appear to be required for midline crossing *in vivo*. A-B) Loss of *Su(dx)* fails to enhance pan neuronal crossing defects in a fra-/- background. A) Stage 15/16 embryos with various combinations of *wild-type* and null *fra* and *Su(dx)* alleles, stained with pan-neuronal marker BP102 (green). Commissures with crossing defects (either thin or completely missing) are indicated with yellow arrowheads. B) Quantification of crossing defects in embryos within the genotypes indicated in A. Percentage of total crossing defects was compared across groups using ANOVA (** p<0.01, **** p<0.0001). N represents number of individual embryos. C-

bioRxiv preprint doi: https://doi.org/10.1101/2023.10.13.562283; this version posted October 17, 2023. The copyright holder for this preprint (which was not certified by peer review) is the author/funder, who has granted bioRxiv a license to display the preprint in perpetuity. It is made available under aCC-BY 4.0 International license.

D) Loss of *Su(dx)* fails to enhance EW crossing defects in a *fra -/-* background. C Stage 15/16 embryos with various combinations of *wild-type* and null *fra* and *nedd4* alleles, expressing GFP under the egl gal4 driver. Embryos are stained with pan-neuronal marker HRP, and GFP to visualize egl neurons. Commissures with EW crossing defects are indicated with arrowheads. D) Quantification of crossing defects in embryos within the genotypes indicated in C. Percentage of crossing defects was compared across groups using ANOVA (** p<0.01, **** p<0.0001). Each data point represents an individual embryo and error bars represent95% confidence intervals around the mean. E-F) Loss of *Su(dx)* fails to enhance EW crossing defects in the fraΔc background. E) Embryos with *Su(dx)* mutations in fraΔc background. F) Quantification of EW crossing defects for the genotypes shown in E. Percentage of crossing defects was compared across groups using ANOVA (** p<0.01, **** p<0.0001). Each data point represents an individual embryo and error bars represent95% confidence intervals around the mean. All scale bars represent 20μM**.**
